# The meaning of a poor childbirth experience – A qualitative phenomenological study with women in Rwanda

**DOI:** 10.1371/journal.pone.0189371

**Published:** 2017-12-08

**Authors:** Judith Mukamurigo, Anna Dencker, Joseph Ntaganira, Marie Berg

**Affiliations:** 1 Institute of Health and Care Sciences, Sahlgrenska Academy, University of Gothenburg, Gothenburg, Sweden; 2 College of Medicine and Health Sciences, School of Public Health, University of Rwanda, Kigali, Rwanda; University of North Carolina at Chapel Hill, UNITED STATES

## Abstract

**Objective:**

Being pregnant and giving birth is a pivotal life event and one that a woman ordinarily remembers for most of her life. A negative childbirth experience can affect a woman’s health well beyond the episode of the labour and birth itself. This study explored the meaning of a poor childbirth experience, as expressed by women who had given birth in Rwanda.

**Methods:**

In a cross-sectional household study conducted in Northern Province and in Kigali City, the capital of Rwanda, a structured questionnaire was answered by women who had given birth one to 13 months earlier. One question, answered by 898 women, asked them to rate their overall experience of childbirth from 0 (very bad) to 10 (very good). Of these, 28 women (3.1%) who had rated their childbirth experience as bad (≤ 4) were contacted for individual interviews. Seventeen of these women agreed to participate in individual in-depth interviews. The texts were analysed with a reflective lifeworld approach.

**Results:**

The essential meaning of a “poor” childbirth experience was that the women had been exposed to disrespectful care, constituted by neglect, verbal or physical abuse, insufficient information, and denial of their husband as a companion. The actions of carers included abandonment, humiliation, shaming and insult, creating feelings of insecurity, fear and distrust in the women. Two of the women did not report any experience of poor care; their low rating was related to having suffered from medical complications.

**Conclusion:**

It is challenging that the main finding is that women are exposed to disrespectful care. In an effort to provide an equitable and high quality maternal health care system in Rwanda, there is a need to focus on activities to implement respectful, evidence-based care for all. One such activity is to develop and provide education programmes for midwives and nurses about professional behaviour when caring for and working with women during labour and birth.

## Introduction

Being pregnant and giving birth to a child is a pivotal life event, comprising an existential change in life values, such as the meaning of life and relation to death [1]. A central part of this is the transition to motherhood, which usually comprises engagement and growth and is especially crucial for a woman having her first child [2].

It has been shown that a negative experience in pregnancy and childbirth has been associated with a lower quality of life, lower self-rated health, persistent negative memory of pain, development of post-traumatic stress disorder, and a persistent fear of childbirth. These in turn have been associated with adverse outcomes such as increased incidence of caesarean birth, postpartum depression and fewer pregnancies in the future [3].

Care offered to women in relation to giving birth varies globally. In Rwanda, the health care system has been developed extensively in the last two decades [4–7]. When it comes to maternity care, women with a normal pregnancy primarily give birth at a health centre (the so-called first level of care), secondly at district hospitals, and thirdly at referral teaching/university hospitals. The statistics show very good progress in terms of maternal health from 1990 to 2015, with a more than 75% reduction in the maternal mortality ratio, from 1071 per 100,000 live births in the year 2000 to 210 in 2015 [8–10]. This can partly be explained by the increase in women’s attendance at antenatal care sessions and an increase in the proportion of women being assisted by a skilled attendant at childbirth [11]. In 2014, 38% of pregnant women completed the four compulsory antenatal standard visits and 90% were assisted at childbirth by a skilled birth attendant [11–15].

In the struggle towards developing good quality and equitable maternal health care systems, there is an urgent need to implement respectful, evidence-based care for all [16]. In relation to this challenge, there is also a need to explore negative experiences and understand the reasons behind them, and whether there are any factors related to health care that can be improved. The aim of the study presented in this paper was to explore the meaning of a poor childbirth experience as expressed by women who had given birth in Rwanda.

## Methods

This study was part of the Maternal Health Research Programme (MatHeR) identifying elements that can improve the quality of maternity care. The research was undertaken by the University of Rwanda in collaboration with the University of Gothenburg and Umeå University in Sweden.

To obtain an understanding of what resulted in poor childbirth experiences for women, a qualitative phenomenological study with a reflective lifeworld research approach was used as it allows the researchers to explore a phenomenon in all its variations of meanings and essential meaning structure [17, 18]. The phenomenon in this study was “a poor childbirth experience as rated by women who had given birth”.

### Setting and participants

A cross-sectional household study was conducted in Northern Province and in Kigali City, the capital of Rwanda, as part of the MatHeR project. The structured questionnaire was answered by 921 women who had given birth one to 13 months earlier. One question asked the women to rate their overall experience of childbirth from 0 (very bad) to 10 (very good). This question was answered by 898 women. Among them, 28 (3.1%) rated their childbirth experience as bad (≤ 4) [19] and were contacted for an individual interview.

### Data collection

Of the 28 eligible women, 7 were not found due to a change in address. The other 21 women were contacted, of which four declined an interview, one directly and three in relation to a booked interview. The remaining 17 women (60%) were individually interviewed once, seven to 18 months (median: 11 months) after the birth of the index child. The interviews were carried out in Kinyarwanda language by the first author (JUM) in a quiet area at the mother’s home with assistance of one research assistant as a note taker. The main characteristics of the participants and their births are shown in Table 1.

**Table 1:** Characteristics of study participants, n = 17.

Characteristic	Median (range) or n (%)
Overall rating of childbirth experience	2 (0–4)
Age, years	30 (21–38)
Parity	
• First child	3 (18)
• Second child	4 (24)
• Third child	7 (41)
• Fourth child	3 (18)
Education level	
• Primary school	13 (76)
• No formal education	4 (24)
Age of index child at interview (months)	11 (7–18)
Presence of companion at health facility	
Yes	13 (76)
No	4 (24)
Place of birth	
• Health centre	6 (35)
• Transferred from health centre to district hospital	9 (53)
• District hospital	2 (12)
Mode of birth	
• Spontaneous vaginal	15 (88)
• Caesarean section	2 (12)
Complications at birth or early after	
• Stillbirth*	3 (18)
• Haemorrhage	2 (12)
• Infection*	2 (12)
• No complication	11 (65)

* One woman had both a stillbirth and an infection

After a short introduction, the mother was asked to describe in detail her overall childbirth experience. Receptive to the women’s narrative, the interviewer posed clarifying questions such as: “Can you describe in more detail? Can you give an example? Please clarify.” The interviews lasted between 36 and 105 minutes, with an average duration of 43 minutes.

### Data analysis

Each interview recorded was transcribed word-by-word (by first author JUM) and translated word-by-word to English by an independent person proficient in English. The analysis was carried out mainly by JUM and MB and preliminary results were discussed several times with AD. First, all the interviews were read through to get a sense of the whole. In further readings, meaning units that answered the research question–the meaning of a poor childbirth experience–were identified and clustered. The following analysis was a continuous slow process of reading and structuring by moving back and forth between parts and the whole, using the research question as the “lens”. A critical reflective stance was held in which presuppositions were restrained in order to be open to the phenomenon showing itself. Gradually the essential meaning of the phenomenon and its structure emerged [18].

### Ethical consideration

The study was approved by the University of Rwanda, School of Public Health Institutional Review Board in May 2014 as Ref: 010/UR/CMHS/SPH/2014 and the National Institute of Statistics Rwanda (0425/2014/10/NISR). Participation was voluntary for all the women. Before the interview, the interviewer informed the participants in detail about the confidentiality of their responses and of their free choice to participate and option to withdraw from the study at any time. Written and signed consent was obtained from all the participants. Verbal authorisation to record the session was asked for before the interview. If anyone had refused, the researchers would have only taken notes, but there were no refusals. Confidentiality was also maintained through conducting the interview in privacy and coding each interview. The women were informed that those in need of any kind of assistance could receive it at a nearby health centre or hospital, which had been informed in advance about the study.

## Results

The essential meaning of a poor childbirth experience was “being exposed to disrespectful care”, constituted by: neglect, verbal and/or physical abuse, insufficient information and denial of the husband as a companion. This meaning pattern was central in 15 out of the 17 women interviewed, and it seemed that being treated poorly by one health care worker was enough to affect the overall childbirth experience negatively for a woman. Women IP 11 and 15 had no poor experience of the care, as such. Their low rating of their childbirth experience was related to suffering medical complications.

In disrespectful care, women’s medical or nursing needs were not adequately fulfilled. Different uncaring actions were present. Care was given in a hierarchical, diminishing and undignified way, including offending, disgracing and shouting, not responding to calls for help, threatening, and condemning. The women were not listened to and they seemed to be punished in various ways. Severe symptoms were ignored, which led to aggravated and sometimes life-threatening conditions.

The exposure to disrespectful care led to several negative consequences for the women, including feelings of fear for themselves and the expected child; shame, sorrow and insecurity; distrust of and loss of confidence in the health care staff; a sense of powerlessness; and avoiding calling for help during the labour process. The absence of a companion or husband seemed to aggravate the sense of powerlessness. Some women, with support from companions, tried to contact another health care facility. Their poor experiences also influenced the women’s choice of health facility in relation to future health care needs.

The following sections provide examples and descriptions of the disrespectful care received. They include quotations using the women’s own narratives and words; the participants are identified by the use of Interview Person code numbers–IP 1, IP 2 and so on.

### Neglect

Neglectful care involves a health care worker not being with the woman or not being with her enough. Being neglected encompassed being ignored or a refusal to listen seriously enough to the woman’s history, actual situation, her wishes or her own conclusions. It comprised an attitude of disinterest, indifference and a lack of empathy. It included not paying attention to a woman’s needs for comfort, not reacting to a call for help or responding only after repeated calls from the woman or her companion. It encompassed health care workers attending on their own terms, being focused on the medical examinations and assessments, and leaving, often without explaining or describing what they had found on examination, or their prognosis for the progress of labour. The nursing comfort measures were mostly absent or minimal and comprised support as basic as providing drinking water after a direct request from the woman. It also comprised neglecting the need for pain relief. The ultimate consequence of being neglected is a feeling of abandonment.

The narrative of IP 16 illustrates neglectful behaviour. When she arrived at the health centre, the maternity health care worker assessed her as being far from the time she would give birth and therefore left her in the waiting room with her mother. As the contractions were increasing during the early morning, the woman called the health worker, who arrived and examined her and concluded that birth was still not imminent, and that it probably would occur later the next day. The woman’s strong contractions continued and her mother was still at her side in the waiting room. They did not call for help, as they felt bad at calling “too early”. The woman gave birth in the waiting room. The health care worker arrived when she heard the child crying and asked the woman, in an accusing way, why they did not call her in time. The woman and her mother explained that it was because she had just told them that it was not time to give birth.

IP 1 was pregnant with her first child when a regular antenatal check-up revealed that the child had died in utero. Labour was induced. A strong sense of being abandoned stands out in her narrative. The health care worker was “behind the curtain”, only came when the woman called, and performed a minimum of medical and nursing care:

*When I was still alone*, *I happened to scream out to the nurse behind the curtain and she did not come*. *I yelled again saying that the baby’s head has come because I was alone and thought it had*. *The nurse came and said “the cervix is yet to open” and left again*. *She told me “continue to push when you feel the contraction”*. *… Feeling exhausted*, *I called the nurse again and asked for something to eat*. *They gave me soda; I drank it and when I finished I handed the bottle to the nurse*, *she put it down and went back away*. *… I went on pushing*, *while alone*, *until the baby’s body came out*. *I called the nurse again and she picked up the body from the delivery table*. (IP 1)

Woman IP 5 had similar experiences of absent health care workers when she gave birth to her second child. No one stayed close by to observe and comfort her; the nurse only arrived when she was called by the woman’s mother-in-law, who was outside but informed about the situation. The woman concluded: *They* (health care workers) *are not near mothers; even when they are expected to give birth*, *they leave you and go out*. (IP 5). IP 12 summarised: *I can’t say anything good about her*. *She didn’t respect me*. *She did not notice my problem*. And IP 7 concluded:

*All we mothers were calling upon her*, *but we could see that she didn’t care*. *She did something only when it came into her mind or when she wanted to*. (IP 7)

Neglect of abnormal obstetric signs was shown in the narrative of IP 3. She had experienced abnormal bleeding during pregnancy, which was aggravated when the contractions started. The health care worker at the maternity unit did not pay attention to this but told the woman to come back when contractions were stronger, despite several complaints. When it was time to give birth, the woman lost consciousness. After the birth, she was sent to the referral hospital where she received blood transfusions.

*I was bleeding a lot before delivery*. *… They sent me back seven times*. *I was unconscious and not able to push the baby… There is no difference between giving birth at home and at the health facility*. (IP3)

### Verbal and/or physical abuse

Being abused comprised mostly verbal, but sometimes physical, abuse. IP 17 arrived at the maternity ward with labour contractions and ruptured amniotic membranes, accompanied by her mother and another woman. The nurse examined her, but talked in an abusive way, shouting and accusing the woman of climbing on the gynaecology table the wrong way. This led to the woman avoiding contacting the nurse during the course of labour and she gave birth without professional assistance:

*When I arrived I met a health professional, he examined me, asking me when the contractions had started, and shouting at me. I felt offended and wondered if this person who shouts like this to me would manage to help me give birth. … I felt desperate and told my mother not to call the nurses anymore and prayed “Oh Lord, you will do what you want to do”. My mother and the other companion supported me until I gave birth on the floor*. *(IP 17)*

IP 10 had been pregnant with her first child and infected with HIV by the child’s father, with whom she now had no contact. She thought the bad behaviour she experienced was related to this. The narrative showed that the nurse repeated insulting words. For example, she asked the woman, “Are you normal?” when the woman, who was being referred because of her HIV-positive status, arrived at the maternity ward. The woman declared:

*I immediately felt hurt inside myself due to the way she communicated with me*. *… There was no respect*. (IP 10)

IP 8 was accompanied by her husband, who assisted with feeding her. The woman had no extra clothes. In addition to neglecting the woman when she called, the health care worker listened instead to music from her phone. The health care worker’s humiliating behaviour was seen in her using the woman’s clothes for cleaning; mocking her; and talking about her situation with other health care workers in a degrading way. IP 7, a primiparous woman, could not stay calm due to severe pain. She asked for pain relief several times:

*Contractions became intense and painful*. *When I called them*, *they intimidated me*, *stating that I am bad and telling me not to shout and that the reason for shouting was that I was still young*. *… that it is childish*. *After having called her four times without getting any support*, *I realised that she would not help me at all*. (IP 7)

IP 9 met a nurse who called the birthing women “goats”:

*I was going there to knock on her* (the nurse’s) *door when she was asleep*. *She was coming*, *very angry*, *saying*, *“But you goats of ladies*, *how are you*, *ladies*?*”… I kept quiet so as not to react to this violent mood even if she insulted me*. (IP 9)

Two of the women (IP 2 and IP 3) had registered for antenatal care late in pregnancy and therefore they had to pay for the childbirth. IP 3 had moved from a neighbouring country. IP 2 had only realised that she was pregnant, very late in the pregnancy. When the labour pains started, she was on her way for her third antenatal check-up. Due to the contractions, she went directly to the labour ward where she was met by the nurse who had done the two earlier pregnancy check-ups:

*I knocked on the door and she asked me*, *“What are you coming to do here without giving me your paper*?*” Looking at me very disdainfully*, *she shouted*, *“Go back*!*” I told her*, *“I am really feeling bad; please do examine me…” I went back and sat there in the room full of other pregnant women and also some husbands*. *I was feeling very bad and insecure and wanted to cry*, *and I had strong contractions…she sent me to a room full of pregnant women and I was ashamed there because she shouted at me instead of comforting me … She diminished me*. (IP 2)

When IP 16 returned for vaccination a week after the birth, the health care worker threatened her by stating that she would be stopped from working if she complained about receiving poor care. IP 5 had an unplanned third pregnancy. Her first child had been stillborn and the second living one was only some months old. The woman’s pregnancy was filled with worries for the young infant and for her own body; she wondered if she would be able to manage. She also had negative memories of health care from earlier births. When she arrived complaining about the painful labour contractions, the following happened:

*They were insulting*, *saying*, *“Behave well or we are going to beat you*, *or we are going to kick you out*, *did you come to give birth or not*? *How many times did you give birth*? *You should not behave like that*.*” … As I did not know what to do*, *they should instead guide me*. (IP 5)

### Insufficiently informed

To be insufficiently informed means not being given information or receiving insufficient information and explanations in relation to what the woman needs, both about what is happening and before actions are taken, such as an examination or a decision to do surgery. The health care workers did not seem to do enough to ensure that the women fully understood the situation, or did not inform them or explain the status and the anticipated progress to them:

*He did not inform me about how far the birth was*, *whether it was a matter of minutes or hours*. *He just told me to find a bed*. (IP 17)

A common example of not getting sufficient information was in relation to conducting a vaginal examination without explanation. This led to a feeling of uncertainty:

*She was doing a vaginal examination to check if the baby had descended*, *and told me that the baby was still high up*. *But she did not tell me what was next*. (IP 7).

Another common situation where information was insufficient was related to performing and suturing an episiotomy. This was the case for IP 6, who described that everything went well at childbirth until a new nurse arrived for the suturing. The nurse did not explain what she was doing, and in addition, denied the woman’s request for pain relief:

*She only came in to carry out her plans* (suturing), *doing her stuff without a word*, *me lying there* (IP 6).

The lack of explanations increased the feeling of being disrespectfully treated:

*I was feeling like a cow they were taking to slaughter*, *when people are looking at things without explaining to you and you don’t see anything*. *When a person doesn’t tell you that you have a certain problem*, *you wonder if you will die or live*. *You think that there is something that they are hiding from you*. (IP 13)

Another example of insufficient explanation happened to IP 14 when the health care workers could not determine the position of her baby during the external examination. They stated that they ‘could not find the head.’ After being told this, the woman and her relatives thought the child’s head was missing, and that the reason was that the mother had been poisoned. After a while, and verified by ultrasound, the child was found to be lying horizontally, in the transverse position, and the head was located. Still, at the time of the interview, the woman was convinced that she had been poisoned:

*“As the baby’s head was missing; so, they have poisoned me…that’s what I was saying and that God had rescued me*, *in case they had poisoned me”,*

### Denial of the husband as a companion

Another type of disrespectful care is refusing to let a woman’s husband be her companion at childbirth. This means that the woman lost a protector. It is common for husbands not to be allowed to be present as they are not considered a “companion” and can therefore not enter the delivery room. Their place was to wait outside. The narrative of IP 16 demonstrates what feelings this causes in the labouring woman:

*When the time to give birth arrived*, *I went to the health centre with my husband*. *They asked why there was no person to care for me and told him to go and look for another female companion*. *It was at eleven p*.*m*. *…*. *I ask myself if they killed him because our home is far away*. (IP 16)

IP 9, who had had been poorly treated by a nurse who spoke abusively, kept quiet and prayed to God for help having the baby. She suggested the following should happen:

*“They should let us give birth in presence of our husbands*. *This strengthens our security.”*

## Discussion

The essential meaning of a poor childbirth as experienced by the women was disrespectful care characterised by neglect, verbal or physical abuse, insufficient information, and denial of the husband as a companion. The actions of carers included abandoning, humiliating, disgracing and insulting the women and creating feelings of insecurity, fear and distrust. The women’s narratives constituted strong evidence of mistreatment, and identifies neglectful care as a legitimate problem needing attention.

Our findings about disrespectful care echo those of extensive research that has been done elsewhere on the African continent. For example, a qualitative study in South Africa exploring factors associated with negative birth experiences found that poor quality intrapartum care led to distress and that it comprised negative interpersonal relations, lack of information, neglect and abandonment, and the absence of a labour companion [20]. Direct observations of maternity care in health care facilities in five countries in Eastern and Southern Africa, of which one was Rwanda, showed that poor interactions between health care providers and the women included two of our four constituents: lack of information and physical and verbal abuse [21].

The denial of spousal participation found in our study is not surprising, as it is more or less a rule in Rwanda to not allow husbands be present during labour and birth of the child [10]. Some of the women believed that if their husband had been present, the disrespectful care would not have occurred. This echoes findings from another study on partner’s participation in maternity care in Rwanda [10]. Denial of companionship by the person a woman wants has been studied elsewhere in the sub-Saharan region [22] and has been described as a crime against humanity [23]. The World Health Organization recently recommended, in suggested intrapartum guidelines, that a parturient woman should be encouraged to have a supportive companion she trusts and can feel at ease with in labour and birth [16]. The denial of the husbands’ presence in our study is partly linked with the fact that many women give birth in the same room in beds separated with curtains. A qualitative study on experiences of men who have attended the birth of their children in Malawi showed that with proper motivational information, a supportive environment, a positive attitude on the part of midwives, and the spouse’s willingness, it is possible to involve male partners during childbirth [24]. As it is compulsory that husbands in Rwanda should be present at the first antenatal care check-up in pregnancy [15], we believe it is possible also to develop strategies that permit them to be present during labour and birth.

A study on behaviours in care in Mali showed that the midwives themselves confirmed that they used disrespectful, abusive care behaviours such as yelling, being insulting, and displaying a hostile or aggressive attitude [25]. Earlier studies have found that the definition of “professional health care,” including during childbirth, is not the same as “good care”. Care has been characterised as being provided on a continuum between the two opposite poles of “caring and uncaring” [26]. The disrespectful care identified in our study corresponds to the negative pole; it is “uncaring”.

We agree with other researchers and policy makers that disrespectful care comprising undignified and humiliating actions needs to be identified in local contexts [27]. An extended literature review has revealed a typology of mistreatment of women during childbirth in health care facilities, concluding that mistreatment can occur at different levels from all the interactions between the woman and provider, through systemic failures at health care facilities, and at the health care system level [28].

According to a systematic literature review on studies in sub-Saharan Africa, disrespectful care is partly related to the governing model of maternity care, which in this region is institution-centred rather than woman-centred. A medico-technical care dominates, and health care workers make efforts to maintain power and control over women’s bodies and knowledge through unsupportive behaviour and being unresponsive to the women’s needs [29], thus providing disrespectful care. In such a medico-technical care system, over-medicalisation is a common practice, with excessive or inappropriate harmful interventions being carried out [16], which often do not take women’s needs and values into consideration. This was obvious in our study. The women experienced neglect as being ignored, such as when they asked for help or pain relief; as a lack of empathy; and as not being believed when they reported the progress of their labour and alarming signs of complications. They experienced lack of empathy and an absence of information and explanations. They experienced abusive behaviours including insulting, punishing words, and sometimes physical abuse.

A landscape analysis in a cross-sectional study in Nigeria has identified seven categories of disrespectful care [30]. Of 446 respondents, 98% had experienced at least one form of disrespectful care. The majority (55%) had been exposed to treatment they had not consented to; 36% had been physically abused; 30% had received undignified care; 29% had been abandoned/neglected; 26% had experienced lack of confidentiality in their care; 22% had been punished in the health facility, and 20% had been discriminated against [23].

An important basis for using a questionnaire with directed questions for studies like this is the recently suggested clinical criteria for respectful evidence-based intrapartum care [16]. Based on research and numerous reports and efforts around the globe, professional associations working together have recently suggested strategies to eliminate disrespectful care at health care facilities. The “mother–baby-friendly birth facility initiative” suggests training, awareness raising, values clarification, supportive supervision, and criteria-based systematic audits of the quality of care measured against a set of criteria [31]. Interventions partly covering these areas are ongoing and have recently been performed in the south Saharan region. In Tanzania, an intervention sought to increase knowledge of patient rights and birth preparedness. However, due to a lack of sufficiently rigorous scientific methodology, it could not draw any strong conclusions about the positive effect identified [32]. Another recent study in 13 facilities in Kenya, with a pre- and post-study design, measured a 7% reduction of disrespect and abuse [4].

## Methodological considerations

The strength of this phenomenological lifeworld study is that it provides further understanding of what it means to have a negative childbirth experience. Furthermore, it is based on a representative sample of Rwandan women who had had a baby within one to13 months. Everyone who rated the childbirth experience as “bad” (≤ 4 out of 10 points) was invited to participate in this study and 60% of them were interviewed [19]. As a result, the findings on the impact of being exposed to disrespectful care could probably be generalised to the study population in our cross-sectional study.

Some limitations in our study need to be clarified. The interviews and the transcripts were in originally in Kinyarwanda, and in translation, words could lose their special meaning. Another limitation is that the women could have interpreted the structured question in the questionnaire about overall experience differently. Furthermore, the experience of childbirth as “poor” could be underreported as definitions of what constitutes a poor childbirth experience could vary significantly and therefore some women who had a poor experience may not have been detected. In addition, some women may have hesitated to give a low rating because they feared that the health care worker providing the childbirth care would be blamed.

## Conclusion

Rwanda is actively striving towards an optimal high-quality health care system. Progress in maternal and child health is being made. However, our study shows that some women are exposed to disrespectful care. Thus, there is need to focus on strategies to implement respectful maternity care for all women. The barriers for health care providers to behave respectfully need to be further investigated, and education programmes for midwives and nurses about professional behaviour should be implemented.
